# Percutaneous Mitral Edge-to-Edge Repair: State of the Art and a Glimpse to the Future

**DOI:** 10.3389/fcvm.2019.00122

**Published:** 2019-09-18

**Authors:** Faisal Khan, Mirjam Winkel, Geraldine Ong, Nicolas Brugger, Thomas Pilgrim, Stephan Windecker, Fabien Praz, Neil Fam

**Affiliations:** ^1^Department of Cardiology, Bern University Hospital, Bern, Switzerland; ^2^Division of Cardiology, St. Michael's Hospital, University of Toronto, Toronto, ON, Canada

**Keywords:** percutaneous mitral repair, mitral valve, mitral regurgitation (MR), MitraClip (MC), transcatheter mitral valve (MV) repair

## Abstract

Patients with severe symptomatic mitral regurgitation have a poor prognosis if left untreated. In those patients who are not eligible for mitral valve surgery, percutaneous edge-to-edge repair may improve clinical outcomes. Recent clinical trials have added to our knowledge and provide interesting insights into the management of such patients. With an increasingly aging global population, these technologies are likely to represent an important treatment option. This mini-review will examine the technology, the evidence and the latest developments in percutaneous mitral edge-to-edge repair.

## Introduction

It has been estimated that nearly 50% of patients with severe symptomatic mitral regurgitation (MR) are not referred for surgery, mainly because of age, and reduced left ventricular function resulting in high surgical risk (1). Conversely, 62% of patients with ischaemic secondary MR and systolic heart failure are dead within 5 years (2). In this light, the MitraClip (MC) mitral valve repair system (Abbott Vascular, Abbott Park, Illinois, USA) has taken center stage as a treatment option, particularly in the context of an aging population. The obvious advantages of a percutaneous approach are reduced invasiveness and rapid recovery. The first procedure was performed in 2003, CE mark obtained in 2008 and FDA approval for the treatment of primary MR in 2013. Transcatheter mitral valve (MV) repair compared with conventional MV surgery has demonstrated similar 5-year mortality in the Endovascular Valve Edge-to-Edge Repair Study II (EVEREST II) albeit at the cost of treatment efficacy compared to surgical MV repair or replacement in patients with predominantly primary MR (3). Surgical treatment of secondary MR is not well established, therefore the recently published results of the randomized MITRA-FR and COAPT trials examining the additive benefits of MC on top of medical therapy specifically in secondary MR populations were highly anticipated (4, 5). While COAPT showed a 47% relative risk reduction of the primary endpoint (all hospitalizations for heart failure at 24 months), as well as a lower mortality after 2 years of follow-up (29.1 vs. 46.1%; hazard ratio, 0.62; 95% CI, 0.46–0.82; *p* < 0.001), no significant difference between groups were found in the smaller MITRA-FR study. These diametrically opposing results can be explained by diverging patient characteristics. This mini-review will examine the technology, the evidence and the latest developments in the field.

## Surgical Treatment

Current European guidelines advocate surgical treatment for symptomatic severe primary MR as a class I indication. Surgery is also recommended in asymptomatic MR in the presence of predictors of worse outcome (atrial fibrillation, left ventricular ejection fraction ≤60%, or LVESD ≥45 mm or systolic pulmonary pressure ≥50 mmHg) or if there is a low surgical risk and a high chance of durable repair in patients with a LVESD ≥40 mm and either a flail leaflet or an enlarged left atrium (6). Although no randomized data are available, surgical repair is preferred over replacement where anatomically possible and is associated with a low recurrence in primary MR (90% of surviving patients after 20 years remain free of severe MR). Observational studies suggest improved clinical outcomes compared with MV replacement (7, 8).

In contrast, surgical repair of secondary MR has less favorable outcomes with increased perioperative mortality and MR recurrence rates as high as 60% within 2 years (9). In patients undergoing mitral-valve repair or replacement for severe ischemic mitral regurgitation, no significant between-group difference in left ventricular reverse remodeling or survival was seen at 2 years. Mitral regurgitation recurred more frequently in the repair group, resulting in more heart-failure–related adverse events and cardiovascular admissions. However, reverse remodeling at 2 years was observed after successful repair rather than replacement (10).

The Alfieri surgical edge to edge repair operation was designed to reduce MR by creating a double orifice from the placement of a stitch joining the free edge of the anterior and posterior mitral valve leaflets (11). The benefit of the operation was effective reduction of MR using a relatively easy and reproducible technique, although it is often combined with annuloplasty for a more durable result.

Despite surgical treatments being available, it is estimated nearly 50% of patients with severe MR are not referred due to prohibitively high risk as a result of age and comorbidity (12). In those older and more comorbid patients undergoing surgical treatments for MR there is generally no increase in long-term survivability and uncertain benefit on quality of life (13). A less invasive treatment option would therefore be particularly appealing for this patient group.

## Percutaneous Mitral Leaflet Repair

The success of transcatheter aortic valve replacement has demonstrated the benefits of innovation in the domain of the treatment of structural heart disease. As this field grows, the next frontiers are effective interventional treatments for the mitral and tricuspid valves. The only percutaneous leaflet repair system with both FDA and CE mark approval is the MitraClip (Abbott, Abbott Park, Illinois). Its competitor, the PASCAL System (Edwards Life sciences, Irvine, California), recently obtained CE mark and a pivotal trial is currently underway aiming for FDA approval. Both systems aim to approximate the mitral valve leaflets to reduce MR.

## Pre-procedural Planning

Pre-procedural planning for MC includes a comprehensive echocardiographic assessment for a precise depiction of the underlying mechanism for regurgitation, as well as grading of MR severity.

On transthoracic echocardiography (TTE), biventricular size and systolic function, left atrium size, other significant valvular disease and estimation of pulmonary pressure based on the Guideline recommended imaging windows and parameters should be obtained (14).

**Figure 1:**
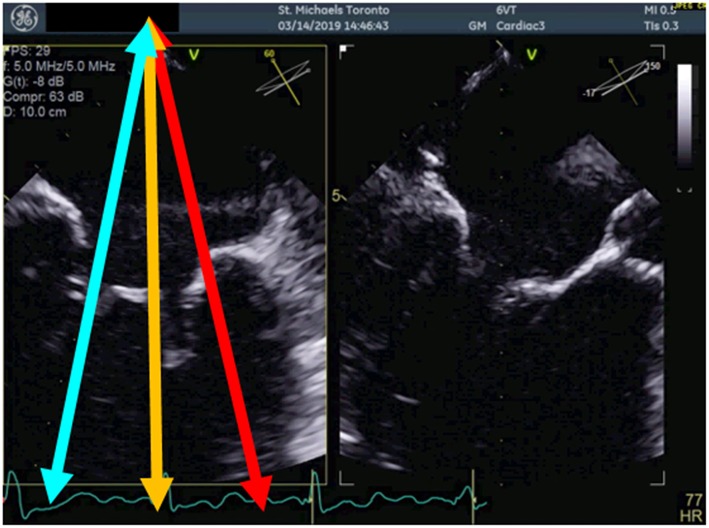
Multiplane view of the mitral valve. Mid-esophageal biplane views (left panel: 60 degree view, right panel: 150 degree view) of the mitral valve leaflets. The blue arrow demonstrates the medial aspect of the mitral valve, the orange arrow, the central aspect and the red arrow, the lateral aspect of the mitral valve.

**Figure 2:**
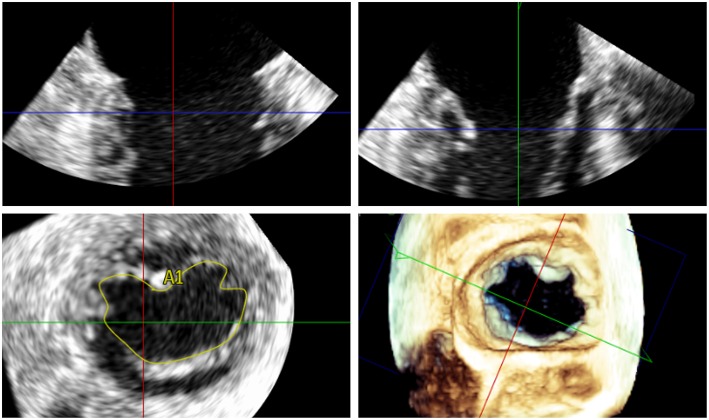
Measurement of the 3D mitral valve area (MVA) by transoesophageal echocardiography in a patient with severe MR; the MVA in this case was 5.48 cm^2^ indicating suitability for percutaneous mitral valve repair.

Transesophageal echocardiography (TEE) is the mainstay for MR intervention screening because of its key role in intraprocedural guidance. A careful examination of the mechanism of MR and quantitative assessment of MR degree of severity should be reported. In addition to the standard 2D echocardiographic views, utilization of advanced imaging is particularly helpful to determine the presence of anatomic abnormality. The use of multiplane imaging allows a systematic visualization of all MV scallops, from the medial to lateral aspects of the MV (Figure 1). An en-face view of the atrial side of the entire MV (surgeon's view) and adjacent structures is possible using 3D imaging. Flail and prolapse segments, the location of clefts, deep indentations, perforations and significant malcoaptation gaps may be more apparent and easier to visualize. In addition, MV area can more precisely be measured (Figure 2).

Current European Guidelines recommend a multiparametric approach for the diagnosis of severe MR including semi-quantitative parameters (vena contracta ≥7 mm, systolic flow reversal in the pulmonary veins, mitral inflow dominant E-wave ≥1.5 m/s, and MR velocity (CW Doppler) TVI mitral/TVI aortic >1.4) and quantitative parameters (effective regurgitant orifice area (EROA) and the regurgitant volume (R Vol), which is ≥ 40 mm^2^ and ≥ 60 ml for primary MR. In secondary MR, an EROA ≥ 20 mm^2^ and R Vol ≥ 30 ml have been shown to have a prognostic value and therefore proposed to indicate severe disease in the European Guidelines, but not in the corresponding Guidelines of the American Society of Echocardiography (2, 6, 15). Quantification of MR severity should be performed using 2D or 3D proximal isovelocity surface area (PISA) method or preferably 3D vena contracta area.

Both TTE and TEE should be reviewed by the Heart Team to confirm eligibility and intraprocedural approach to MV repair. Agreement should be made as to the precise location for device placement, number of device, and treatment strategies, particularly with more challenging anatomy as defined in Table 1.

**Table 1:** Anatomical considerations for percutaneous mitral leaflet repair.

**Favorable**	**Unfavorable/contraindicated**
Moderate-severe or severe MR	Commissural lesions
A2-P2 defect	Clefts
Prolapse width <15 mm	Short posterior leaflet (<5 mm)
Flail gap <10 mm	Mitral valve orifice area < 3.5 cm^2^
Mitral valve orifice area > 4 cm^2^	Severe calcification of grasping zone
Mobile length of the posterior leaflet ≥ 7 mm	Leaflet perforations
	Mitral stenosis with mean gradient≥5 mmHg at baseline
	Active endocarditis or rheumatic heart disease

## Abbott MitraClip: The Procedure

The MitraClip device has been implanted in over 100,000 patients worldwide. It is introduced percutaneously via a 24 French orientable guiding catheter from the femoral vein using a superior and posterior trans-septal puncture to access the left atrium. A steerable clip delivery catheter enables orientation of the clip whilst real-time 3D transoesophageal echo allows precision targeting of the free edges of the opposing leaflets at the site of regurgitation. The device is then advanced into the left ventricle and while pulling back the catheter, the mitral valve leaflets are grasped. Once optimal grasping has been undertaken, the clip is closed creating a double orifice. Transoesophageal echo is used to assess for adequate leaflet insertion, residual MR and new trans-valvular gradients avoiding mitral stenosis prior to final deployment. Direct measurement of LA mean pressure and V wave provides complementary hemodynamic data to guide treatment decision-making. Multiple clips can be implanted to optimize imperfect results on a case by case basis if gradients and anatomy allow. Hemodynamics usually remain very stable during the procedure and recovery time is short.

## Newest Iteration: MitraClip XTR

There is currently a new version of the clip, the MitraClip XTR (MC XTR), which is similar to the first generation and NT versions of the MC in that it consists of a 24 French steerable guide catheter and a steerable clip delivery system (CDS). The MC XTR has a 5 mm longer clip grasping width due to longer arms (22 vs. 17 mm compared with the NTR). The transition zone between the delivery sheath and the CDS has been reinforced to improve stability during rotation of the CDS. The steerable sleeve is also more responsive to the rotation of the M-knob. The working length of the system has been increased by 1.5 cm and changes to the mechanism and material of the lock line enable operation of the system in the “unlocked” position. Finally a new Nitinol rather than Elgiloy gripper line enables a deeper gripper drop and grasping angle. Ultimately, the MC XTR may enable easier and quicker leaflet grasping, reduce the number of clips required and expand percutaneous treatment to patients with less favorable anatomy.

**Figure 3:**
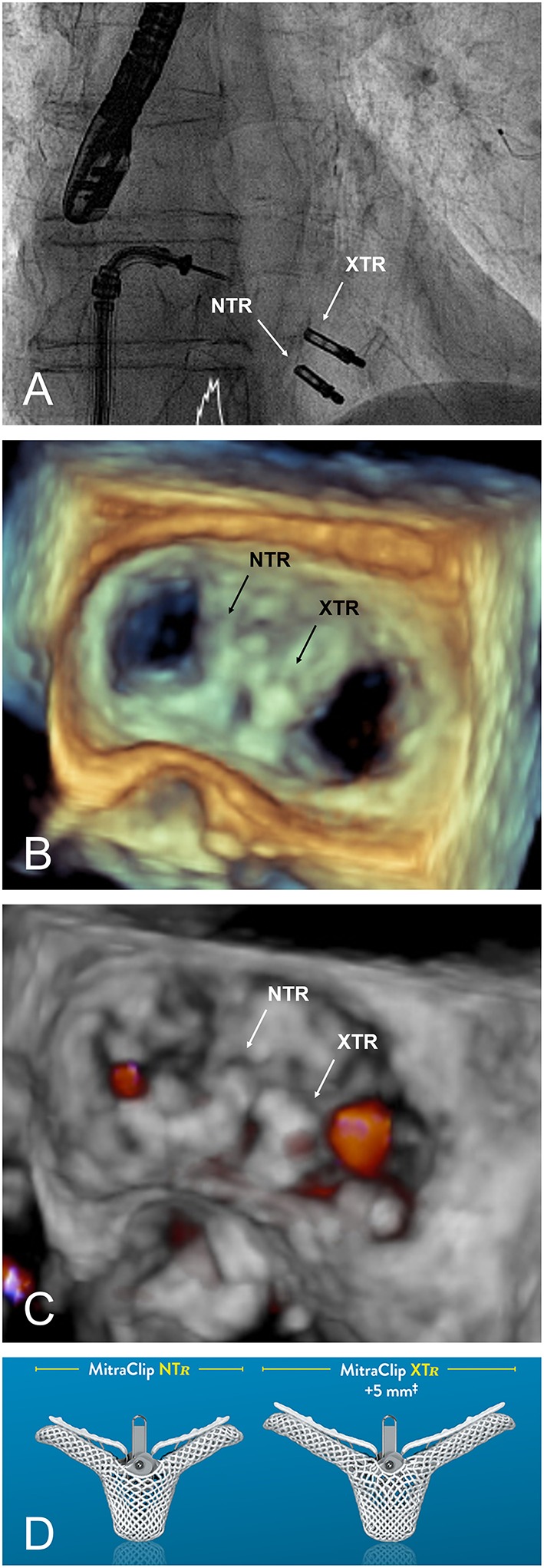
**(A)** Fluoroscopic appearance of a successfully implanted XTR and NTR clip. **(B,C)** 3D transoesophageal echocardiographic view and Doppler, respectively, providing an en face view of the valve from the same case after implantation. **(D)** MitraClip versions currently available.

On the other hand, grasping more tissue may result in additional tension on the leaflets that concentrates at the tip of the clip arms. This may provoke leaflet damage, especially in patients with calcifications, fragile, or thin appearing leaflets. Moreover, due to the increased length of the clip arms, the risk of entrapment in the subvalvular apparatus is certainly higher, particularly when treating commissural lesions. According to a recently published multicenter experience in 107 patients treated with the MC XTR, procedural success was high with MR≤2+ in 93% of the patients and ≤1+ in 77%. However, four patients had leaflet damage requiring surgical correction during the same hospitalization (16). Thus, the use of the XTR system should be evaluated based on individual anatomy, rather than as a default strategy. Although requiring further evaluation, the combination of different clip sizes may represent a valuable treatment option in patients on whom valve area/gradient is borderline (Figure 3). It is anticipated that future iterations of the MitraClip will allow independent leaflet grasping.

## The Edwards PASCAL Mitral Repair System (Edwards Lifesciences, Irvine, CA, USA)

**Figure 4:**
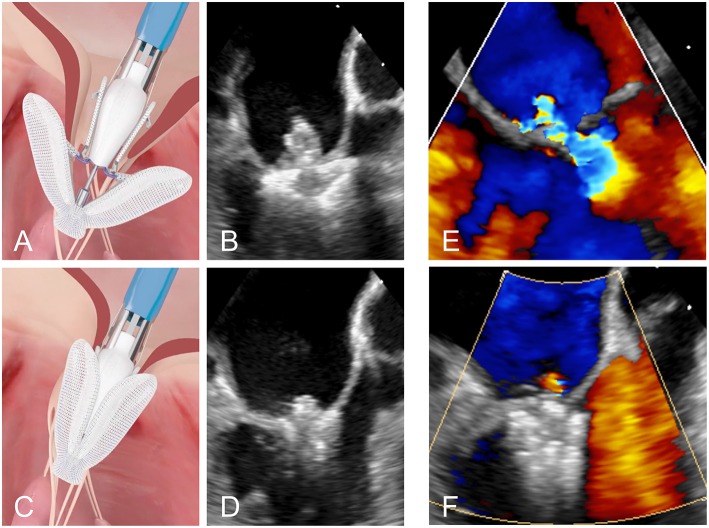
**(A,B)** Positioning of the PASCAL device in the mitral valve. **(C,D)** Grasping of the two leaflets. **(E,F)** Transoesophageal Doppler image of a case of severe MR before and after leaflet repair with the PASCAL device, respectively.

The Edwards PASCAL Transcatheter Valve Repair System has been designed to address some of the limitations of previous systems. It is intended to reduce the tension on the valve leaflets by introducing a 10 mm central spacer within the MV regurgitant orifice. The paddles of the implant are also wider and curved to further reduce tension and the system allows for independent grasping of the leaflets. This may be particularly useful in the presence of a large prolapse gap (Figure 4) or in patients with retraction or tethering of the posterior leaflet. The device is also designed to be easier navigated in the left atrium and offers a higher degree of steerability. A first-in-human feasibility experience of the device has been described from a series of 23 compassionate use cases (17). These early data were encouraging with MR ≤2+ in 97% of the patients at discharge and without elevated gradients despite a larger device size.

The CLASP study is a multicenter, prospective trial of the PASCAL system in 62 patients with significant MR despite medical therapy, with independent adjudication of clinical events and central echo core lab. Mean age was 76.5 years, NYHA class II/IV in 51.6%, with 56% FMR, 36% DMR, and 8% mixed MR etiology. At 30 days, the major adverse event rate was 6.5%, with all-cause mortality of 1.6% (18). Overall, 98% of patients had MR ≤ 2+, and 86% had MR ≤ 1+, with 85% in NYHA Class I/II; significant improvements were also observed in 6 min walk distance and KCCQ scores. Based on these promising results, the PASCAL system gained CE mark in early 2019. The pivotal CLASP IID/F randomized trial has begun enrolment, and will compare the efficacy and safety of PASCAL vs. MitraClip in patients with significant DMR or FMR, using a non-inferiority study design.

## Evidence From Randomized Trials

EVEREST II (NCT00209274, Endovascular Valve Edge-to-Edge REpair Study) was the first randomized trial to examine the MitraClip system in 279 patients with moderate-to-severe or severe MR, comparing percutaneous therapy to conventional surgery in a respective 2:1 ratio (19). Published in 2011, this was a very early experience with the system for many of the recruiting sites. The percutaneous intervention arm demonstrated superior safety with similar improvements in clinical outcomes although was less effective at reducing MR compared to surgery at 1 year. The primary end point for efficacy (freedom from death, MV surgery, reintervention, and moderate-to-severe MR) was 55% in the MitraClip group and 73% in the surgical group at 12 months (*p* = 0.007). Major adverse events occurred in 15% of patients in the MitraClip group and 48% of patients in the surgical group at 30 days (*p* < 0.001). The 5 year follow-up from this study found the composite endpoint in the as-treated population was 44.2 vs. 64.3% in the percutaneous repair and surgical groups, respectively (*p* = 0.01) driven primarily by more MR and more subsequent mitral surgery in the percutaneous arm. Rates of surgery and moderate-to-severe MR were comparable between groups beyond 6 months, affirming the durability of both techniques. Notably, only 27% of the patients in this trial had secondary MR.

The French MITRA-FR study randomized 307 patients with symptomatic left ventricular dysfunction and significant secondary MR to either medical therapy or medical therapy combined with the MitraClip procedure (5). 92% of patients achieved an MR grade ≤2+ immediately after the procedure while there was no difference in the primary outcome of all-cause death and unplanned re-hospitalization for heart failure at 1 year which occurred in 55% of the intervention group and 51% of the control group (odds ratio [OR], 1.16; 95% confidence interval [CI] 0.73–1.84; *p* = 0.53). The mortality rate was 24.3% in the intervention group vs. 22.4% in the control group (hazard ratio [HR], 1.11; 95% CI 0.69–1.77) at 12 months. As an important limitation, it has to be mentioned that a significant amount of follow-up data on echocardiographic outcome and functional status at 12 months were missing.

The presentation of the results from MITRA-FR were closely followed by the North American COAPT trial which randomized 614 patients with symptomatic heart failure and moderate-to-severe or severe secondary MR to medical therapy or medical therapy and MitraClip repair (4). The primary outcome was the rate of hospitalization for heart failure within 24 months which was 35.8% per patient-year in the device group as compared with 67.9% in the control group (HR, 0.53; 95% CI, 0.40–0.70; *p* < 0.001). Moreover, the powered secondary end point of death from any cause within 24 months was significantly lower occurring in 29.1% of the patients in the device group as compared with 46.1% in the control group (HR, 0.62; 95% CI, 0.46–0.82; *p* < 0.001). The number of patients needed to treat to prevent 1 hospitalization was 3 and to prevent 1 death was 6. All prespecified secondary endpoints including quality of life and functional assessments were significantly improved in the MitraClip arm.

While both trials examining MC in the context of secondary MR produced different results, there were major differences between the two trials. Firstly, due to differing definitions of severe functional mitral regurgitation between European and North American guidelines, mitral regurgitation was more severe in the COAPT trial than in the MITRA-FR trial (mean EROA of 41 vs. 31 mm^2^). In addition, the indexed left ventricular end-diastolic volumes were smaller in COAPT as compared with MITRA-FR (101 ± 34 vs. 135 ± 35 ml/m2). Another difference between the trials may have been increased aggressiveness of the guideline directed medical therapy delivered to the patients in COAPT which was overseen by the screening committee. Taken together, this might mean the patients in COAPT had worse MR with relatively more preserved left ventricles representing a group a patients that benefit most from percutaneous edge-to-edge repair.

Secondly the number of patients receiving more than one clip was higher in COAPT possibly explaining the higher proportion of moderate-to-severe or severe residual MR at 1 year in MITRA-FR (17 vs. 5% in COAPT).

## Future Directions

There are a number of questions still remaining with regards to percutaneous leaflet repair:

1. Empirical use of antiplatelet therapy for stroke prevention after the procedure has been advocated without any trial evidence. Recent registry data suggests the use of a NOAC with a single antiplatelet may prove beneficial as compared with antiplatelet therapy alone, especially in the first year post implantation (20). Randomized data would be needed to more accurately define the answer to this important question.
2. The evaluation of percutaneous edge-to-edge repair in cases of cardiogenic shock resulting from acute MR would be an interesting avenue to explore, particularly in the setting of subacute myocardial infarction where surgical repair remains hazardous.
3. The objective echocardiographic grading of post-procedural residual MR is very challenging and requires further validation.
4. The management of the atrial septal defect created during MitraClip has to be further clarified.
5. Continuous left atrial pressure monitoring is a promising but still not well-standardized method to evaluate outcome of repair.
6. Further data is required to understand what is the place of the PASCAL device within treatment options and whether it can tackle complex anatomy such as larger flail segments, shorter posterior leaflets, or cases involving mitral annular calcification; this may be answered by a future head to head trial.

The development of newer devices and iterations in the field of percutaneous leaflet repair may expand the spectrum of anatomies that can be treated. Patients with primary MR and favorable anatomy who are inoperable or at high risk for surgery, can reasonably be offered percutaneous mitral valve repair. In secondary MR, patient selection seems to be of paramount importance to optimize individual outcomes. Volume overload from excessive secondary MR should no longer be thought of as an innocent bystander but rather a contributing factor to poor outcomes in patients with heart failure. Based on the data available, guideline directed medical therapy for heart failure should be optimized with cardiac resynchronization where appropriate prior to consideration of percutaneous mitral valve repair. If despite these measures symptomatic moderate-severe or severe functional MR remains, an early approach to treatment should be considered before further deterioration of ventricular performance occurs. The early detection and appropriate management of these patients in a multidisciplinary Heart Team is crucial to allow timely interventional treatment. Identification of factors predicting response to the therapy is expected to be a topic of future research. Potential meaningful parameters may include the proportionality of MR related to ventricular dilation, the presence of myocardial fibrosis precluding ventricular remodeling, as well as the use of strain echocardiography to better appreciate myocardial function (21).

While new device iterations allow novel features such as independent grasping or increasing arm dimensions, they also introduce new challenges such as possible asymmetric grasping resulting in residual MR or excessive leaflet tension and thus require a further learning curve for optimal use.

With the recent advances in technology and an expanding knowledge base from carefully conducted randomized clinical trials, we are already glimpsing into a future where percutaneous therapies have an important role in the management of mitral valve disease and heart failure.

## Author Contributions

FK, MW, GO, FP, and NF: drafting of the manuscript and figures. NB: figures and critical revision of the content. SW and TP: critical revision of the content.

### Conflict of Interest Statement

TP has received research grants to the institution from Edwards Lifesciences, Symetis/Boston Scientific, and Biotronik, and speaker fees from Boston Scientific and Biotronik. FP has served as a consultant for Edwards Lifesciences. SW has received research and educational grants from Abbott, Amgen, BMS, Bayer, Boston Scientific, Biotronik, CSL, Medtronic, Edwards, Sinomed, and Polares. NF is a Consultant for Edwards Lifesciences, Speaker for Abbott and Proctor for NaviGate Cardiac Structures Inc. The remaining authors declare that the research was conducted in the absence of any commercial or financial relationships that could be construed as a potential conflict of interest.
